# Supplementary material for: Causal Relationship between Aging and Anorexia Nervosa: A White-Matter-Microstructure-Mediated Mendelian Randomization Analysis
Source: Biomedicines. 2024 Aug 16;12(8):1874. doi: 10.3390/biomedicines12081874 (PMC11351342; doi:10.3390/biomedicines12081874)
Supplement: Supplementary file 1 [file biomedicines-12-01874-s001.zip › Supplementary Figure/Figure S4/Singe SNP plot of MR analysis for TL to ubm-b-1680.pdf]

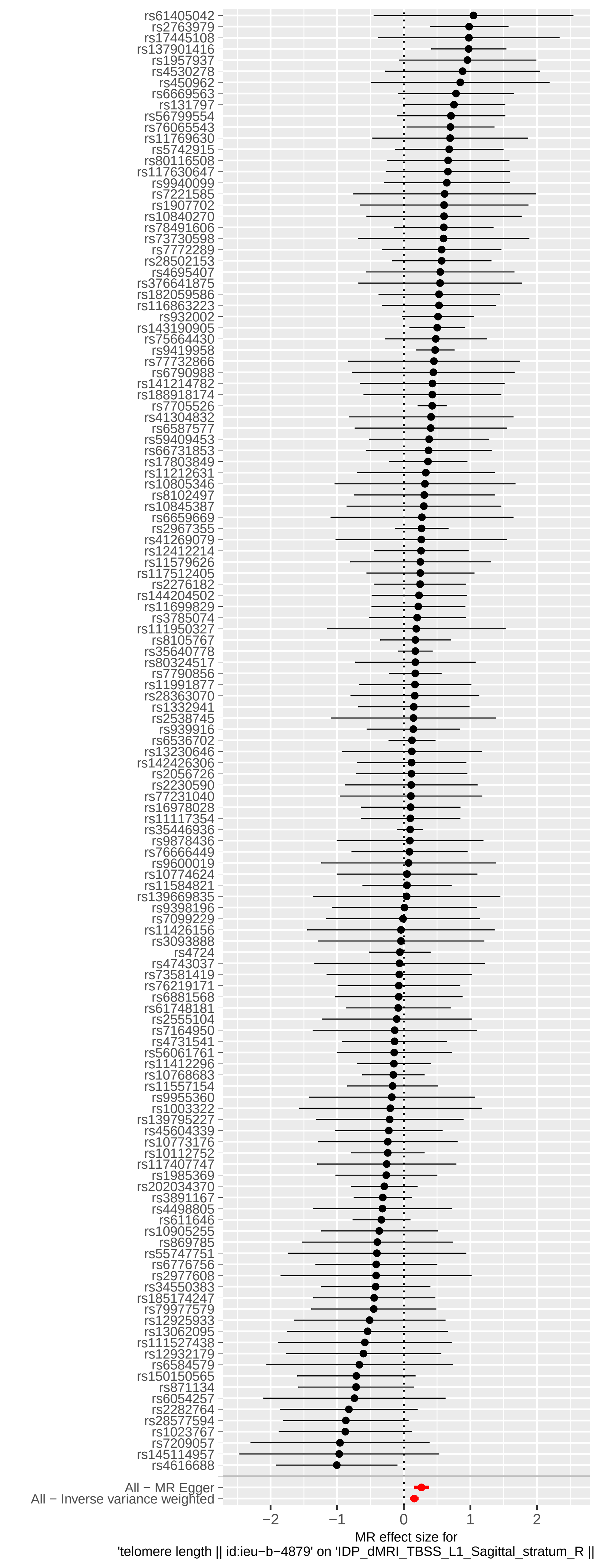
rs61405042
rs2763979
rs17445108
rs137901416
rs1957937
rs4530278
rs450962
rs6669563
rs131797
rs56799554
rs76065543
rs11769630
rs5742915
rs80116508
rs117630647
rs9940099
rs7221585
rs1907702
rs10840270
rs78491606
rs73730598
rs7772289
rs28502153
rs4695407
rs376641875
rs182059586
rs116863223
rs932002
rs143190905
rs75664430
rs9419958
rs77732866
rs6790988
rs141214782
rs188918174
rs7705526
rs41304832
rs6587577
rs59409453
rs66731853
rs17803849
rs11212631
rs10805346
rs8102497
rs10845387
rs6659669
rs2967355
rs41269079
rs12412214
rs11579626
rs117512405
rs2276182
rs144204502
rs11699829
rs3785074
rs111950327
rs8105767
rs35640778
rs80324517
rs7790856
rs11991877
rs28363070
rs1332941
rs2538745
rs939916
rs6536702
rs13230646
rs142426306
rs2056726
rs2230590
rs77231040
rs16978028
rs11117354
rs35446936
rs9878436
rs76666449
rs9600019
rs10774624
rs11584821
rs139669835
rs9398196
rs7099229
rs11426156
rs3093888
rs4724
rs4743037
rs73581419
rs76219171
rs6881568
rs61748181
rs2555104
rs7164950
rs4731541
rs56061761
rs11412296
rs10768683
rs11557154
rs9955360
rs1003322
rs139795227
rs45604339
rs10773176
rs10112752
rs117407747
rs1985369
rs202034370
rs3891167
rs4498805
rs611646
rs10905255
rs869785
rs55747751
rs6776756
rs2977608
rs34550383
rs185174247
rs79977579
rs12925933
rs13062095
rs111527438
rs12932179
rs6584579
rs150150565
rs871134
rs6054257
rs2282764
rs28577594
rs1023767
rs7209057
rs145114957
rs4616688
All − MR Egger
All − Inverse variance weighted
−2
−1
0
1
2
MR effect size for
'telomere length || id:ieu−b−4879' on 'IDP_dMRI_TBSS_L1_Sagittal_stratum_R ||
